# 2-Amino-5-bromo­pyridine–benzoic acid (1/1)

**DOI:** 10.1107/S1600536810005969

**Published:** 2010-02-20

**Authors:** Madhukar Hemamalini, Hoong-Kun Fun

**Affiliations:** a X-ray Crystallography Unit, School of Physics, Universiti Sains Malaysia, 11800 USM, Penang, Malaysia

## Abstract

In the title adduct, C_5_H_5_BrN_2_·C_7_H_6_O_2_, the carboxyl group of the benzoic acid mol­ecule is twisted away from the attached ring by 12.97 (11)°. The 2-amino-5-bromo­pyridine mol­ecules inter­act with the carboxylic group of neighbouring benzoic acid mol­ecules through N—H⋯O and O—H⋯N hydrogen bonds, forming cyclic *R*_2_^2^(8) hydrogen-bonded motifs and linking the mol­ecules into a two-dimensional network lying parallel to (100). The crystal structure is further stabilized by weak C—H⋯O hydrogen bonds.

## Related literature

For background to the chemistry of substituted pyridines, see: Pozharski *et al.* (1997 ▶); Katritzky *et al.* (1996 ▶). For related structures, see: Goubitz *et al.* (2001 ▶); Vaday & Foxman (1999 ▶). For details of hydrogen bonding, see: Jeffrey & Saenger (1991 ▶); Jeffrey (1997 ▶); Scheiner (1997 ▶). For hydrogen-bond motifs, see: Bernstein *et al.* (1995 ▶); For bond-length data, see: Allen *et al.* (1987 ▶). For the stability of the temperature controller used in the data collection, see: Cosier & Glazer (1986 ▶).
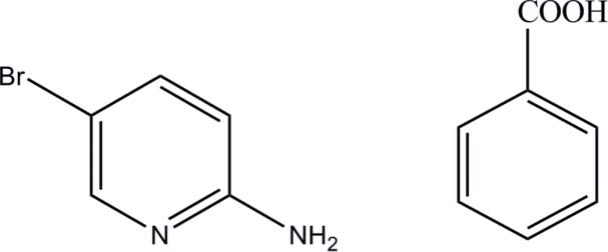


## Experimental

### 

#### Crystal data

C_5_H_5_BrN_2_·C_7_H_6_O_2_
*M* *_r_* = 295.14
Monoclinic, 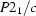

*a* = 18.5614 (16) Å
*b* = 5.1769 (5) Å
*c* = 12.3613 (11) Å
β = 97.016 (2)°
*V* = 1178.91 (19) Å^3^
*Z* = 4
Mo *K*α radiation
μ = 3.48 mm^−1^
*T* = 100 K
0.61 × 0.21 × 0.07 mm

#### Data collection

Bruker APEX DUO CCD area-detector diffractometer
Absorption correction: multi-scan (*SADABS*; Bruker, 2009 ▶) *T*_min_ = 0.228, *T*_max_ = 0.788
19825 measured reflections
5495 independent reflections
3709 reflections with *I* > 2σ(*I*)
*R*_int_ = 0.057

#### Refinement

*R*[*F* ^2^ > 2σ(*F* ^2^)] = 0.045
*wR*(*F* ^2^) = 0.123
*S* = 1.01
5495 reflections
155 parameters
H-atom parameters constrained
Δρ_max_ = 1.25 e Å^−3^
Δρ_min_ = −0.50 e Å^−3^

### 

Data collection: *APEX2* (Bruker, 2009 ▶); cell refinement: *SAINT* (Bruker, 2009 ▶); data reduction: *SAINT*; program(s) used to solve structure: *SHELXTL* (Sheldrick, 2008 ▶); program(s) used to refine structure: *SHELXTL*; molecular graphics: *SHELXTL* software used to prepare material for publication: *SHELXTL* and *PLATON* (Spek, 2009 ▶).

## Supplementary Material

Crystal structure: contains datablocks global, I. DOI: 10.1107/S1600536810005969/rz2420sup1.cif

Structure factors: contains datablocks I. DOI: 10.1107/S1600536810005969/rz2420Isup2.hkl

Additional supplementary materials:  crystallographic information; 3D view; checkCIF report

## Figures and Tables

**Table 1:** Hydrogen-bond geometry (Å, °)

*D*—H⋯*A*	*D*—H	H⋯*A*	*D*⋯*A*	*D*—H⋯*A*
O1—H1⋯N1^i^	0.82	1.83	2.626 (2)	162
N2—H2*A*⋯O2^ii^	0.86	2.02	2.866 (3)	167
N2—H2*B*⋯O1^iii^	0.86	2.25	3.105 (2)	171
C7—H7⋯O2^iv^	0.93	2.51	3.064 (2)	118

Symmetry codes: (i) 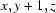; (ii) 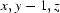; (iii) 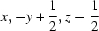; (iv) 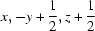.

**Fig. 1.**
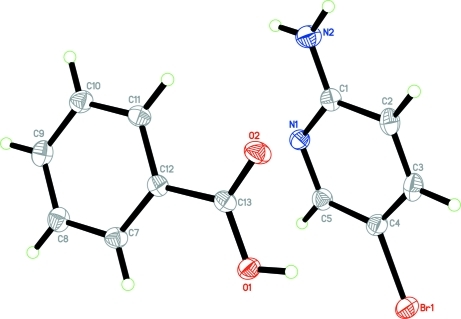
The asymmetric unit of the title compound. Displacement ellipsoids are drawn at the 50% probability level.

**Fig. 2.**
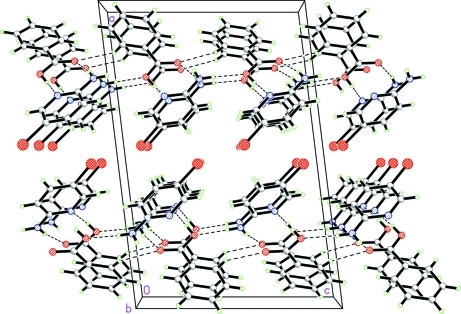
The crystal packing of the title compound, showing hydrogen-bonded (dashed lines) networks.

## supplementary crystallographic information

### Comment

Pyridine and its derivatives play an important role in heterocyclic chemistry (Pozharski *et al.*, 1997; Katritzky *et al.*, 1996). They are often involved in hydrogen-bond interactions (Jeffrey & Saenger, 1991; Jeffrey, 1997; Scheiner, 1997). The crystal structures of 2-amino-5-bromopyridine (Goubitz *et al.*, 2001) and 2-amino-5-bromopyridinium propynoate (Vaday & Foxman, 1999) have been reported in the literature. In the present study, the hydrogen-bonding patterns in the 2-amino-5-bromopyridine benzoic acid (1/1) cocrystal are investigated.

The asymmetric unit (Fig 1), contains one 2-amino-5-bromopyridine molecule and one benzoic acid molecule. The 2-amino-5-bromopyridine molecule is planar, with a maximum deviation of 0.024 (2)Å for atom N2. The carboxyl group of the benzoic acid molecule is twisted away from the attached ring by 12.97 (11)° . The bond lengths (Allen *et al.*, 1987) and angles are normal.

In the crystal packing (Fig. 2), the 2-amino-5-bromopyridine molecules interact with the carboxylic group of the respective benzoic acid molecules through N2—H2A···O2 and O1—H1···N1 hydrogen bonds, forming a cyclic hydrogen-bonded motif *R*_2_^2^(8) (Bernstein *et al.*, 1995), and linking the molecules into 2-dimensional networks parallel to the (100) plane. The crystal structure is further stabilized by strong N2—H2B···O1 and weak C7—H7···O2 (Table 1) hydrogen bonds.

### Experimental

A hot methanol solution (20 ml) of 2-amino-5-bromopyridine (87 mg, Aldrich) and benzoic acid (61 mg, Merck) were mixed and warmed over a heating magnetic stirrer for a few minutes.  The resulting solution was allowed to cool slowly at room temperature and crystals of the title compound appeared after a few days.

### Refinement

All hydrogen atoms were positioned geometrically [C–H = 0.93 Å, N–H = 0.86 Å and O–H = 0.82 Å] and were refined using a riding model, with *U*_iso_(H) = 1.2 *U*_eq_(C, N) or 1.5 *U*_eq_(O).

### Crystal data

**Table:**

C_5_H_5_BrN_2_·C_7_H_6_O_2_	*F*(000) = 592
*M**_r_* = 295.14	*D*_x_ = 1.663 Mg m^−^^3^
Monoclinic, *P*2_1_/*c*	Mo *K*α radiation, λ = 0.71073 Å
Hall symbol: -P 2ybc	Cell parameters from 3648 reflections
*a* = 18.5614 (16) Å	θ = 3.8–32.0°
*b* = 5.1769 (5) Å	µ = 3.48 mm^−^^1^
*c* = 12.3613 (11) Å	*T* = 100 K
β = 97.016 (2)°	Plate, colourless
*V* = 1178.91 (19) Å^3^	0.61 × 0.21 × 0.07 mm
*Z* = 4	

### Data collection

**Table:**

Bruker APEX DUO CCD area-detector diffractometer	5495 independent reflections
Radiation source: fine-focus sealed tube	3709 reflections with *I* > 2σ(*I*)
graphite	*R*_int_ = 0.057
φ and ω scans	θ_max_ = 35.9°, θ_min_ = 3.8°
Absorption correction: multi-scan (*SADABS*; Bruker, 2009)	*h* = −30→30
*T*_min_ = 0.228, *T*_max_ = 0.788	*k* = −8→8
19825 measured reflections	*l* = −20→20

### Refinement

**Table:**

Refinement on *F*^2^	Primary atom site location: structure-invariant direct methods
Least-squares matrix: full	Secondary atom site location: difference Fourier map
*R*[*F*^2^ > 2σ(*F*^2^)] = 0.045	Hydrogen site location: inferred from neighbouring sites
*wR*(*F*^2^) = 0.123	H-atom parameters constrained
*S* = 1.01	*w* = 1/[σ^2^(*F*_o_^2^) + (0.064*P*)^2^] where *P* = (*F*_o_^2^ + 2*F*_c_^2^)/3
5495 reflections	(Δ/σ)_max_ = 0.001
155 parameters	Δρ_max_ = 1.25 e Å^−^^3^
0 restraints	Δρ_min_ = −0.50 e Å^−^^3^

### Special details

**Table:**

Experimental. The crystal was placed in the cold stream of an Oxford Cryosystems Cobra open-flow nitrogen cryostat (Cosier & Glazer, 1986) operating at 100.0 (1) k.
Geometry. All s.u.'s (except the s.u. in the dihedral angle between two l.s. planes) are estimated using the full covariance matrix. The cell s.u.'s are taken into account individually in the estimation of s.u.'s in distances, angles and torsion angles; correlations between s.u.'s in cell parameters are only used when they are defined by crystal symmetry. An approximate (isotropic) treatment of cell s.u.'s is used for estimating s.u.'s involving l.s. planes.
Refinement. Refinement of F^2^ against ALL reflections. The weighted R-factor wR and goodness of fit S are based on F^2^, conventional R-factors R are based on F, with F set to zero for negative F^2^. The threshold expression of F^2^ > 2σ(F^2^) is used only for calculating R-factors(gt) etc. and is not relevant to the choice of reflections for refinement. R-factors based on F^2^ are statistically about twice as large as those based on F, and R- factors based on ALL data will be even larger.

### Fractional atomic coordinates and isotropic or equivalent isotropic displacement parameters (Å^2^)

**Table:**

	*x*	*y*	*z*	*U*_iso_*/*U*_eq_	
Br1	0.470134 (11)	0.30630 (5)	0.886018 (18)	0.02683 (8)	
N1	0.31375 (10)	−0.1799 (3)	0.73265 (14)	0.0191 (3)	
N2	0.25884 (11)	−0.2217 (4)	0.55605 (15)	0.0252 (4)	
H2A	0.2364	−0.3558	0.5758	0.030*	
H2B	0.2519	−0.1704	0.4894	0.030*	
C1	0.30452 (10)	−0.0928 (4)	0.62906 (16)	0.0188 (3)	
C2	0.34283 (11)	0.1260 (4)	0.59759 (17)	0.0214 (4)	
H2	0.3351	0.1861	0.5262	0.026*	
C3	0.39160 (12)	0.2490 (4)	0.67336 (19)	0.0231 (4)	
H3	0.4172	0.3930	0.6543	0.028*	
C4	0.40175 (11)	0.1516 (4)	0.78013 (17)	0.0210 (4)	
C5	0.36211 (11)	−0.0591 (4)	0.80675 (16)	0.0206 (4)	
H5	0.3688	−0.1204	0.8780	0.025*	
O1	0.23842 (7)	0.4732 (3)	0.82571 (10)	0.0188 (3)	
H1	0.2668	0.5550	0.7929	0.028*	
O2	0.19345 (9)	0.3546 (3)	0.65709 (11)	0.0217 (3)	
C7	0.13808 (11)	0.1436 (4)	0.91317 (15)	0.0186 (4)	
H7	0.1616	0.2660	0.9600	0.022*	
C8	0.09178 (11)	−0.0370 (4)	0.95201 (16)	0.0215 (4)	
H8	0.0842	−0.0341	1.0250	0.026*	
C9	0.05710 (11)	−0.2199 (4)	0.88304 (18)	0.0215 (4)	
H9	0.0265	−0.3401	0.9097	0.026*	
C10	0.06795 (12)	−0.2244 (4)	0.77347 (18)	0.0217 (4)	
H10	0.0446	−0.3477	0.7269	0.026*	
C11	0.11352 (10)	−0.0450 (4)	0.73404 (15)	0.0191 (3)	
H11	0.1205	−0.0473	0.6608	0.023*	
C12	0.14884 (10)	0.1389 (4)	0.80347 (15)	0.0163 (3)	
C13	0.19580 (10)	0.3321 (4)	0.75609 (15)	0.0163 (3)	

### Atomic displacement parameters (Å^2^)

**Table:**

	*U*^11^	*U*^22^	*U*^33^	*U*^12^	*U*^13^	*U*^23^
Br1	0.02372 (11)	0.02522 (13)	0.03212 (12)	−0.00568 (8)	0.00565 (8)	−0.00818 (9)
N1	0.0222 (7)	0.0160 (8)	0.0196 (7)	−0.0029 (6)	0.0045 (6)	−0.0009 (6)
N2	0.0331 (9)	0.0235 (9)	0.0182 (7)	−0.0074 (8)	0.0000 (7)	0.0037 (7)
C1	0.0201 (8)	0.0154 (8)	0.0212 (8)	0.0012 (7)	0.0041 (7)	0.0016 (7)
C2	0.0221 (8)	0.0194 (9)	0.0239 (8)	0.0008 (7)	0.0076 (7)	0.0056 (8)
C3	0.0208 (8)	0.0181 (9)	0.0320 (10)	−0.0006 (7)	0.0097 (8)	0.0032 (8)
C4	0.0191 (8)	0.0176 (9)	0.0268 (9)	−0.0005 (7)	0.0053 (7)	−0.0045 (7)
C5	0.0220 (8)	0.0194 (9)	0.0208 (8)	−0.0005 (8)	0.0043 (7)	0.0011 (7)
O1	0.0209 (6)	0.0193 (7)	0.0159 (6)	−0.0052 (6)	0.0013 (5)	0.0003 (5)
O2	0.0310 (7)	0.0199 (7)	0.0145 (5)	−0.0032 (6)	0.0043 (5)	0.0000 (5)
C7	0.0219 (8)	0.0183 (9)	0.0153 (7)	−0.0007 (7)	0.0010 (6)	0.0010 (7)
C8	0.0225 (8)	0.0226 (10)	0.0199 (8)	−0.0018 (8)	0.0041 (7)	0.0052 (8)
C9	0.0207 (8)	0.0165 (9)	0.0277 (9)	−0.0007 (7)	0.0043 (7)	0.0054 (8)
C10	0.0218 (8)	0.0168 (9)	0.0266 (9)	−0.0009 (7)	0.0027 (7)	−0.0033 (8)
C11	0.0214 (8)	0.0171 (9)	0.0190 (8)	0.0013 (7)	0.0033 (6)	−0.0029 (7)
C12	0.0176 (7)	0.0141 (8)	0.0173 (7)	0.0018 (6)	0.0031 (6)	0.0008 (6)
C13	0.0191 (8)	0.0143 (8)	0.0158 (7)	0.0022 (7)	0.0028 (6)	0.0002 (6)

### Geometric parameters (Å, °)

**Table:**

Br1—C4	1.887 (2)	O1—H1	0.8200
N1—C1	1.349 (3)	O2—C13	1.225 (2)
N1—C5	1.355 (3)	C7—C8	1.394 (3)
N2—C1	1.339 (3)	C7—C12	1.395 (2)
N2—H2A	0.8600	C7—H7	0.9300
N2—H2B	0.8600	C8—C9	1.380 (3)
C1—C2	1.417 (3)	C8—H8	0.9300
C2—C3	1.377 (3)	C9—C10	1.394 (3)
C2—H2	0.9300	C9—H9	0.9300
C3—C4	1.404 (3)	C10—C11	1.384 (3)
C3—H3	0.9300	C10—H10	0.9300
C4—C5	1.378 (3)	C11—C12	1.391 (3)
C5—H5	0.9300	C11—H11	0.9300
O1—C13	1.317 (2)	C12—C13	1.493 (3)
			
C1—N1—C5	118.98 (18)	C8—C7—H7	120.3
C1—N2—H2A	120.0	C12—C7—H7	120.3
C1—N2—H2B	120.0	C9—C8—C7	120.53 (18)
H2A—N2—H2B	120.0	C9—C8—H8	119.7
N2—C1—N1	118.04 (19)	C7—C8—H8	119.7
N2—C1—C2	120.74 (19)	C8—C9—C10	120.01 (19)
N1—C1—C2	121.21 (19)	C8—C9—H9	120.0
C3—C2—C1	119.56 (19)	C10—C9—H9	120.0
C3—C2—H2	120.2	C11—C10—C9	119.9 (2)
C1—C2—H2	120.2	C11—C10—H10	120.1
C2—C3—C4	118.38 (19)	C9—C10—H10	120.1
C2—C3—H3	120.8	C10—C11—C12	120.26 (18)
C4—C3—H3	120.8	C10—C11—H11	119.9
C5—C4—C3	119.6 (2)	C12—C11—H11	119.9
C5—C4—Br1	120.26 (16)	C11—C12—C7	119.95 (18)
C3—C4—Br1	120.10 (16)	C11—C12—C13	118.04 (16)
N1—C5—C4	122.22 (19)	C7—C12—C13	121.98 (18)
N1—C5—H5	118.9	O2—C13—O1	123.04 (18)
C4—C5—H5	118.9	O2—C13—C12	120.31 (18)
C13—O1—H1	109.5	O1—C13—C12	116.65 (16)
C8—C7—C12	119.37 (19)		
			
C5—N1—C1—N2	177.14 (19)	C7—C8—C9—C10	−0.3 (3)
C5—N1—C1—C2	−1.8 (3)	C8—C9—C10—C11	−0.1 (3)
N2—C1—C2—C3	−177.5 (2)	C9—C10—C11—C12	0.4 (3)
N1—C1—C2—C3	1.4 (3)	C10—C11—C12—C7	−0.3 (3)
C1—C2—C3—C4	0.1 (3)	C10—C11—C12—C13	−178.42 (19)
C2—C3—C4—C5	−1.2 (3)	C8—C7—C12—C11	0.0 (3)
C2—C3—C4—Br1	178.02 (16)	C8—C7—C12—C13	177.99 (19)
C1—N1—C5—C4	0.6 (3)	C11—C12—C13—O2	12.0 (3)
C3—C4—C5—N1	0.9 (3)	C7—C12—C13—O2	−166.00 (19)
Br1—C4—C5—N1	−178.36 (15)	C11—C12—C13—O1	−168.26 (18)
C12—C7—C8—C9	0.3 (3)	C7—C12—C13—O1	13.7 (3)

### Hydrogen-bond geometry (Å, °)

**Table:**

*D*—H···*A*	*D*—H	H···*A*	*D*···*A*	*D*—H···*A*
O1—H1···N1^i^	0.82	1.83	2.626 (2)	162
N2—H2A···O2^ii^	0.86	2.02	2.866 (3)	167
N2—H2B···O1^iii^	0.86	2.25	3.105 (2)	171
C7—H7···O2^iv^	0.93	2.51	3.064 (2)	118

Symmetry codes: (i) *x*, *y*+1, *z*; (ii) *x*, *y*−1, *z*; (iii) *x*, −*y*+1/2, *z*−1/2; (iv) *x*, −*y*+1/2, *z*+1/2.
